# The Burden of Nephrotoxic Drug Prescriptions in Patients with Chronic Kidney Disease: A Retrospective Population-Based Study in Southern Italy

**DOI:** 10.1371/journal.pone.0089072

**Published:** 2014-02-18

**Authors:** Ylenia Ingrasciotta, Janet Sultana, Francesco Giorgianni, Achille Patrizio Caputi, Vincenzo Arcoraci, Daniele Ugo Tari, Claudio Linguiti, Margherita Perrotta, Andrea Nucita, Fabio Pellegrini, Andrea Fontana, Lorenzo Cavagna, Domenico Santoro, Gianluca Trifirò

**Affiliations:** 1 Department of Clinical and Experimental Medicine, University of Messina, Messina, Italy; 2 Caserta Local Health Service, Caserta, Italy; 3 Department of Cognitive Science, Educational and Cultural Studies (CSECS), University of Messina, Messina, Italy; 4 Unit of Biostatistics, IRCCS "Casa Sollievo della Sofferenza", San Giovanni Rotondo (FG), Italy; 5 Division of Rheumatology, University and IRCCS Foundation Policlinico S. Matteo, Pavia, Italy; National Cancer Institute, United States of America

## Abstract

**Background:**

The use of nephrotoxic drugs can further worsening renal function in chronic kidney disease (CKD) patients. It is therefore imperative to explore prescribing practices that can negatively affect CKD patients.

**Aim:**

To analyze the use of nephrotoxic drugs in CKD patients in a general population of Southern Italy during the years 2006–2011.

**Methods:**

The general practice “Arianna” database contains data from 158,510 persons, registered with 123 general practitioners (GPs) of Caserta. CKD patients were identified searching: CKD-related ICD-9 CM codes among causes of hospitalization; CKD-relevant procedures undergone in hospital (e.g. dialysis); drug prescriptions issued for a CKD-related indication. A list of nephrotoxic drugs was compiled and validated by pharmacologists and nephrologists. The summary of product characteristics was used to classify drugs as ‘contraindicated’ or ‘to be used with caution’ in renal diseases. Frequency of nephrotoxic drug use, overall, by drug class and single compounds, by GPs within one year prior or after first CKD diagnosis and within one year after dialysis entry was calculated.

**Results:**

Overall, 1,989 CKD patients and 112 dialysed patients were identified. Among CKD patients, 49.8% and 45.2% received at least one prescription for a contraindicated nephrotoxic drug within one year prior or after first CKD diagnosis, respectively. In detail, 1,119 CKD patients (56.3%) had at least one nonsteroidal anti-inflammatory drugs (NSAIDs) prescription between CKD diagnosis and end of follow-up. A large proportion of CKD patients (35.6%) were treated with NSAIDs for periods exceeding 90 days. Contraindicated nephrotoxic drugs were used commonly in CKD, with nimesulide (16.6%) and diclofenac (11.0%) being most frequently used.

**Conclusions:**

Contraindicated nephrotoxic drugs were highly prescribed in CKD patients from a general population of Southern Italy. CKD diagnosis did not seem to reduce significantly the prescription of nephrotoxic drugs, which may increase the risk of preventable renal function deterioration.

## Introduction

Chronic kidney disease (CKD) is a progressive and widely prevalent disorder worldwide. In the past decade, prevalence of CKD has doubled in the general population [1]. Prevalence rates of moderate CKD (eGFR <60 ml/min per 1.73 m^2^) were reported to range from 0.2% in 20–39 year-olds to 24.9% in >70 year-olds in population-based studies from America [2], and similarly from Italy [3].

End stage renal disease (ESRD) requiring dialysis or kidney transplantation is a frequent outcome in patients with CKD stage 3 and 4 [4]. Over the last decade, the number of CKD patients requiring dialysis has increased annually by 6.1% in Canada [5], 11% in Japan [6] and 9% in Australia [7]. CKD may progress toward ESRD which results in a significant reduction of patient and relatives quality of life due to increasing morbidity and disability, in addition to increasing healthcare costs [8]. These observations underline the urgent need for strategies to prevent renal diseases [9]. Worsening of renal function is often due to the use (especially long term use at high dosage) of nephrotoxic drugs such as nonsteroidal anti-inflammatory drugs (NSAIDs) [10]–[12].

Nephrotoxic drugs should therefore be avoided or used with caution in patients with underlying CKD. Most of the drugs known to be nephrotoxic exert their toxic effects through different pathogenic mechanisms, such as altered intraglomerular hemodynamics, tubular cell toxicity, inflammation, crystal nephropathy, rhabdomyolysis, and thrombotic microangiopathy [13], [14]. No population-based studies in Italy investigated the use of nephrotoxic drugs in CKD patients so far.

This study is aimed at addressing this research gap by exploring the use of nephrotoxic drugs in patients with CKD in a general population of Southern Italy in the years 2006–2011.

## Methods

### Data Source

Data were extracted from the Arianna database from the years 2005–2011. This database was set up by the Caserta Local Health Agency in Southern Italy in the year 2000 and currently contains information on a population of almost 400,000 inhabitants who are registered in the list of almost 300 general practitioners (GPs). Participating GPs record data during their daily clinical practice using dedicated software and send complete and anonymous clinical data of their patients to the Arianna Database on a monthly basis. The Arianna database can be linked to a hospital discharge registry through a unique and anonymous patient identifier. Quality and completeness of data out of the defined ranges were investigated and back-submitted to each participating GP in order to receive an immediate feedback. GPs failing to meet these standard quality criteria were excluded from the epidemiologic surveys according to the basic standards in the conduction of pharmacoepidemiological investigations. Of all the GPs in Caserta, 123 GPs covering a population of 158,510 inhabitants met these standard quality criteria for the considering period.

Information collected included patient demographics, prescriptions for drugs (coded according to the Anatomical Therapeutic Chemical classification system (ATC)) reimbursed by National Health System and their indications for use, and hospital admissions and procedures (coded by the ninth edition of International Classification of Diseases, Clinical Modification (ICD-9 CM)). So far, the Arianna database has been shown to provide accurate and reliable information for pharmacoepidemiological research, as documented elsewhere [15]–[21].

### Nephrotoxic drugs assessment

Nephrotoxic drugs were identified through a stepwise procedure. A literature review was conducted by using specific MeSH terms ‘nephrotoxic drug’ and ‘drug-induced renal failure’, identifying the most relevant publications. The resulting literature review yielded a list of 127 potentially nephrotoxic drugs which was validated by two nephrologists (DS, VC) and two clinical pharmacologists (GT, VA). Finally, based on the Summary of Product Characteristic (SPC), we classified all these drugs as “contraindicated drugs” (see Table S1) or “drugs to be used with caution (i.e., precaution of use)” in renal diseases (see Table S2). For each contraindicated nephrotoxic drug we identified the specific contraindication as reported in the SPC (see Table S3).

### Study population

For the evaluation of the nephrotoxic drugs prescription in CKD patients, we included all patients with a first-ever diagnosis of CKD during the study period 2006–2011 and with at least one year of database history prior to study entry. The year 2005 was considered as a run-in period to identify newly diagnosed CKD patients from 2006 onwards. The index date (ID) was defined as the date of first CKD diagnosis. We identified CKD patients as those with at least one of the specific codes among either primary/secondary causes of hospital admission, procedures or indication of use associated to the prescribed drugs (see Table S4). Of these, we also identified those requiring dialysis (see Table S4).

### Statistical analysis

The assessment of the exposure to contraindicated drugs and drugs to be used with caution was carried out on all patients with incident CKD (N = 1,989, 1.25% of the total study population). We analyzed the frequency of CKD patients who received at least one prescription of nephrotoxic drug during three different time intervals, i.e. within one year prior and within one year after the first CKD diagnosis as well as within one year after entry in dialysis.

The frequency of CKD patients receiving at least one prescription of nephrotoxic drug by drug classes, was explored in the same three time intervals. Since NSAIDs were the most frequently prescribed drugs, a post-hoc frequency analysis by single compound for this class only was carried out. As low-dose acetylsalicylic acid (aspirin) is contraindicated only in severe renal disease even though it is frequently prescribed for cardiovascular prevention in CKD patients, we analyzed this drug separately.

A sensitivity analysis on the frequency of a limited number of CKD patients (identified by stage-specific codes) receiving at least one prescription of nephrotoxic drug was carried out.

Baseline CKD patients’ characteristics were reported as mean and standard deviation (SD) and frequencies (percentages) for continuous and categorical variables, respectively. Age and sex adjusted multilevel logistic regressions [22] were performed to evaluate the proportion of CKD patients receiving at least one contraindicated nephrotoxic drug within one year prior and after CKD diagnosis. Multilevel methods allowed to appropriately model within- and between-patient variability [23], accounting for the multilevel nature of the data (patients clustered within GP), and to control simultaneously for the possible confounding effects of the different variables (e.g. patient’s case-mix). Posterior means for the proportion of contraindicated nephrotoxic drug prescriptions, provided by each GP, were estimated from random intercepts, controlling for level-one patient’s age and sex centered variables, and were graphically represented along with the observed frequencies of drug prescriptions.

To identify predictors of short- (< 6 months) and long-term (≥ 6 months) NSAIDs use, an ordinary and cumulative age-sex adjusted logistic regression models were performed using non users for NSAIDs as comparator. Cumulative logistic regressions models the patient’s probability of being treated with NSAIDs during the entire follow-up (<180 days or more). Possible predictors of NSAID use, evaluated any time prior to the date of first registration of CKD diagnosis, were also considered. The risk of receiving short and long term therapy was reported as odds ratio (OR), along with their 95% confidence interval (95%CI). Two-sided p-values <0.05 were considered for statistical significance. All the analyses were performed using SAS Release 9.3 (SAS Institute, Cary, NC).

## Results

**Figure 1:**
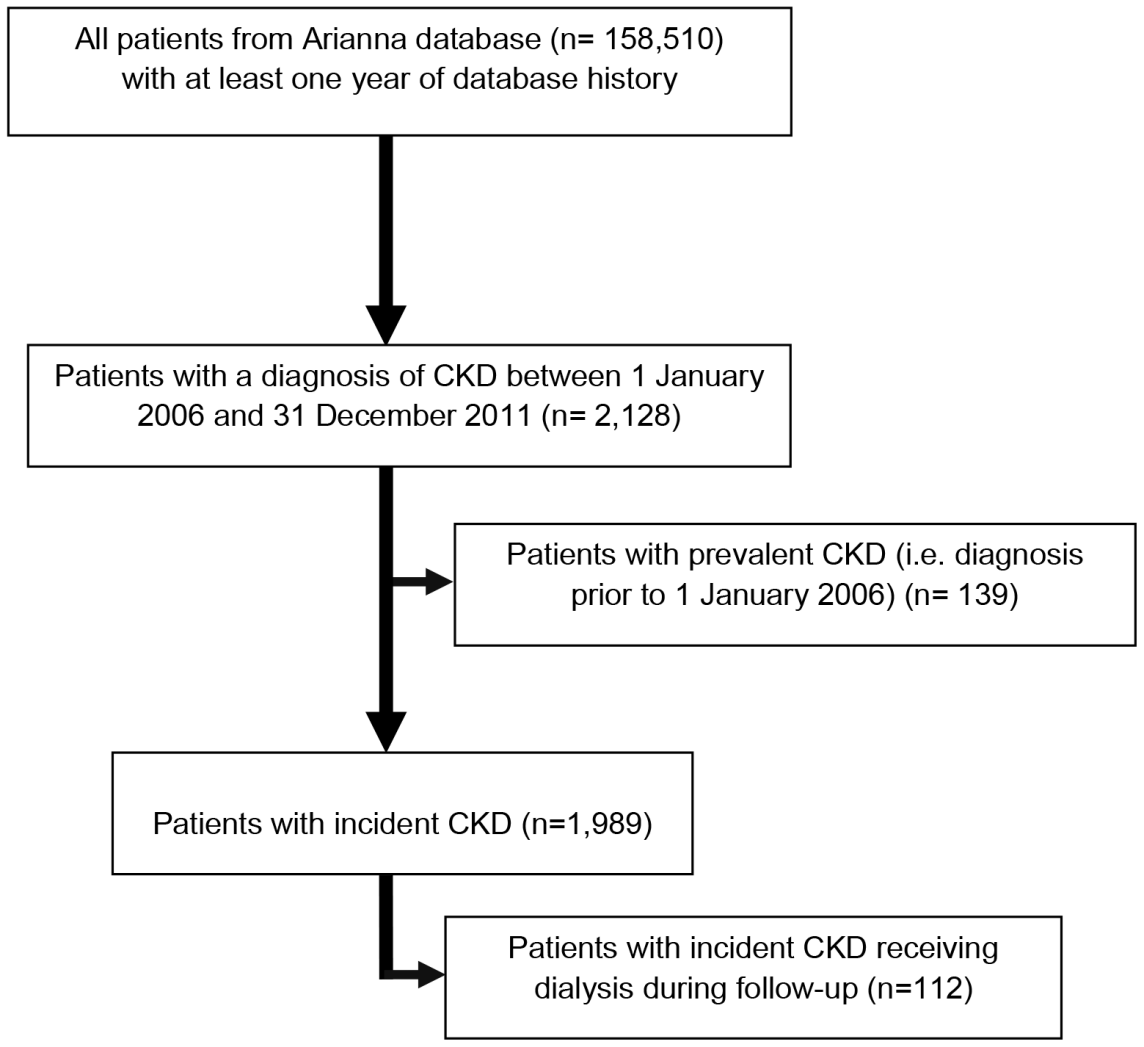
Flow chart of CKD patients included in the study.

**Figure 2:**
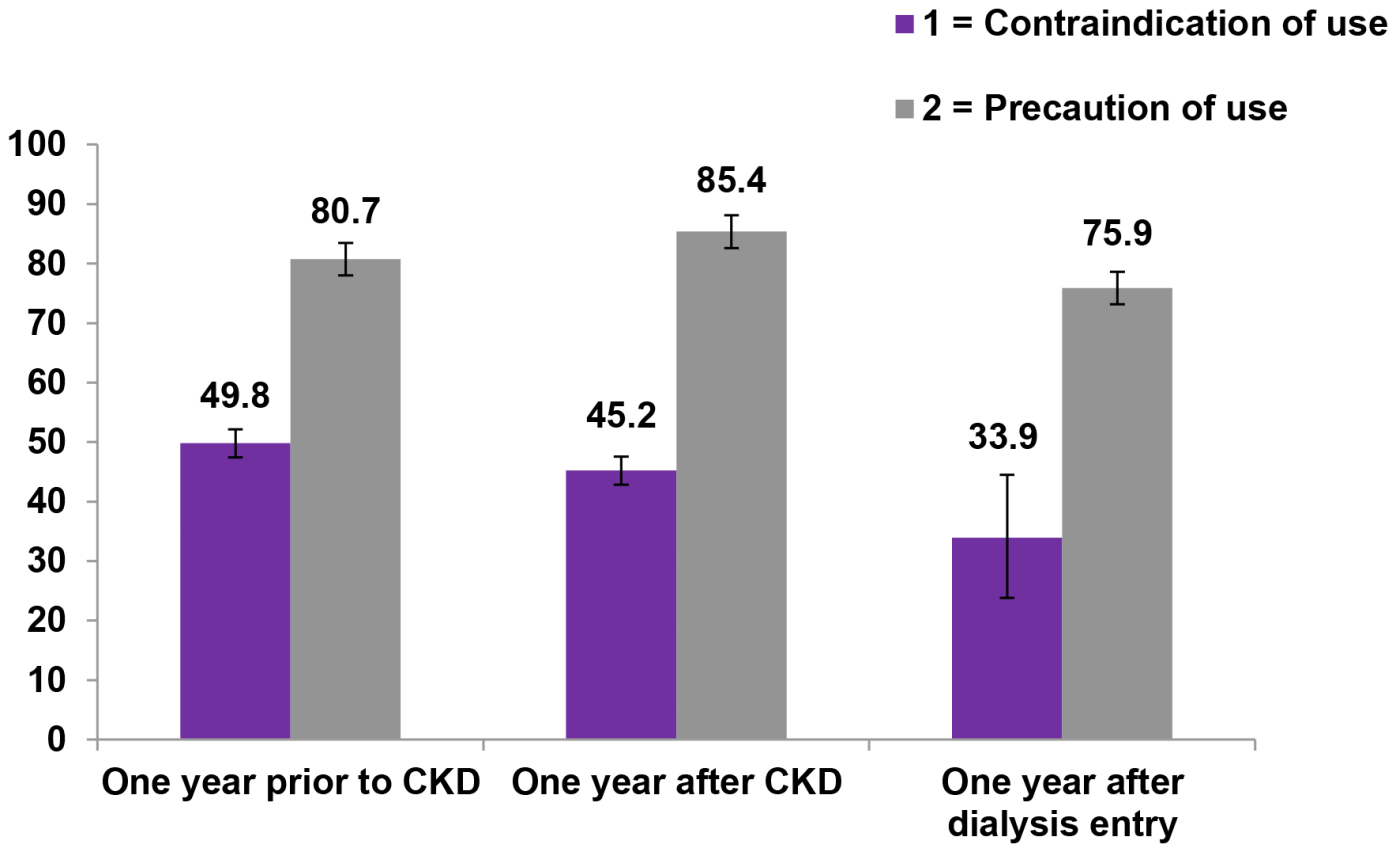
Proportion of incident CKD patients (N = 1,989) who received at least one prescription of nephrotoxic drug. Proportion of incident CKD patients (N = 1,989) who received at least one prescription of nephrotoxic drug within one year prior or after first CKD diagnosis, and within one year after dialysis entry (N = 112).

A cohort of 2,128 patients with both prevalent and incident CKD was identified during the years 2006–2011. Of these, we included in the analysis 1,989 newly diagnosed CKD patients (1.25% of the total general population). Among incident CKD patients, 112 (5.6%) underwent dialysis during the observation period (Figure 1). The mean follow-up time of CKD patients between the index date and end of follow-up was 2.6 years. Figure 2 shows that 49.8% of incident CKD patients (N = 1,989) received at least one prescription for a contraindicated nephrotoxic drug within one year prior to the first CKD diagnosis with only a slight decrease of this proportion (45.2%) within one year after CKD diagnosis. A slightly lower proportion (33.9%) of CKD patients treated with contraindicated drugs was observed within one year after dialysis entry.

Overall, 41.2% of incident CKD patients received more than 5 different contraindicated nephrotoxic drugs and 64.6% of them received more than 5 prescriptions of these drugs during the entire follow-up. Drugs that should be used with caution in CKD were generally more commonly prescribed, with an increase in the proportion of patients receiving at least one prescription after the CKD diagnosis, as compared to the year prior to the diagnosis (85.4% vs. 80.7%). During the entire follow-up after CKD diagnosis, allopurinol (39.7%) and ramipril (26.7%) were the most commonly prescribed drugs to be used with caution.

**Figure 3:**
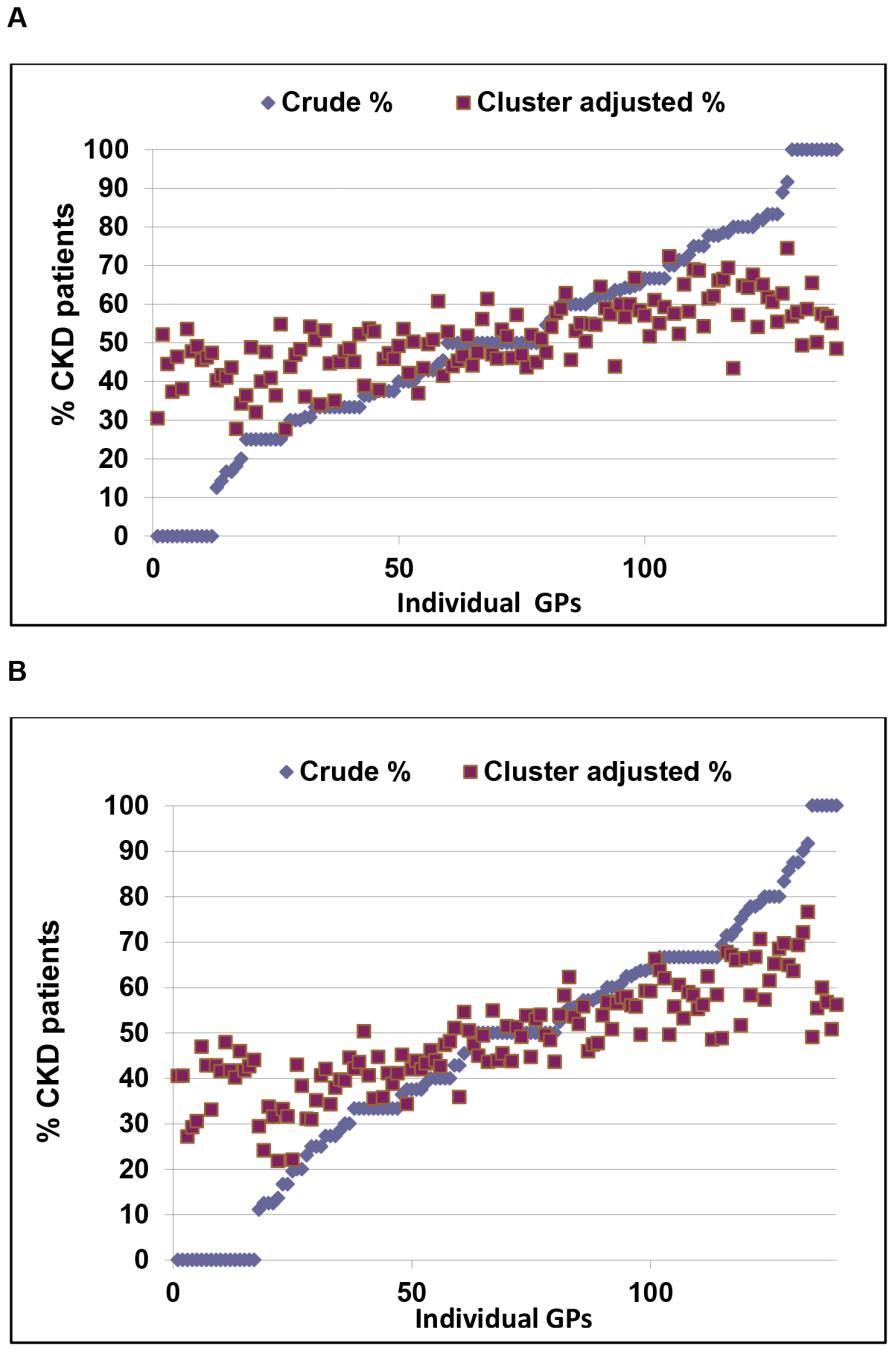
Crude and Cluster-adjusted analysis of CKD patients’ CND prescription by GP. Distribution (%) of CKD patients who received at least one prescription of contraindicated nephrotoxic drugs (CND) within the year prior (A) and after (B) first CKD diagnosis, clustered by GP. The distribution was clustered by GP and the was adjusted also by sex and age. CND: contraindicated nephrotoxic drug.

The range of CKD patients who received at least one prescription of contraindicated nephrotoxic drugs from each individual GP, varied from 0 to 100%. Of note, 3 GPs continued to prescribe at least one contraindicated nephrotoxic drug to 100% of their CKD patients also within one year after CKD diagnosis. The cluster-adjusted results showed that all GPs prescribed nephrotoxic drugs to 27.7–74.5% of their patients before the CKD diagnosis and 21.8–76.6% after the CKD diagnosis, suggesting that the CKD diagnosis did not significantly alter the prescribing pattern (Figure 3).

**Figure 4:**
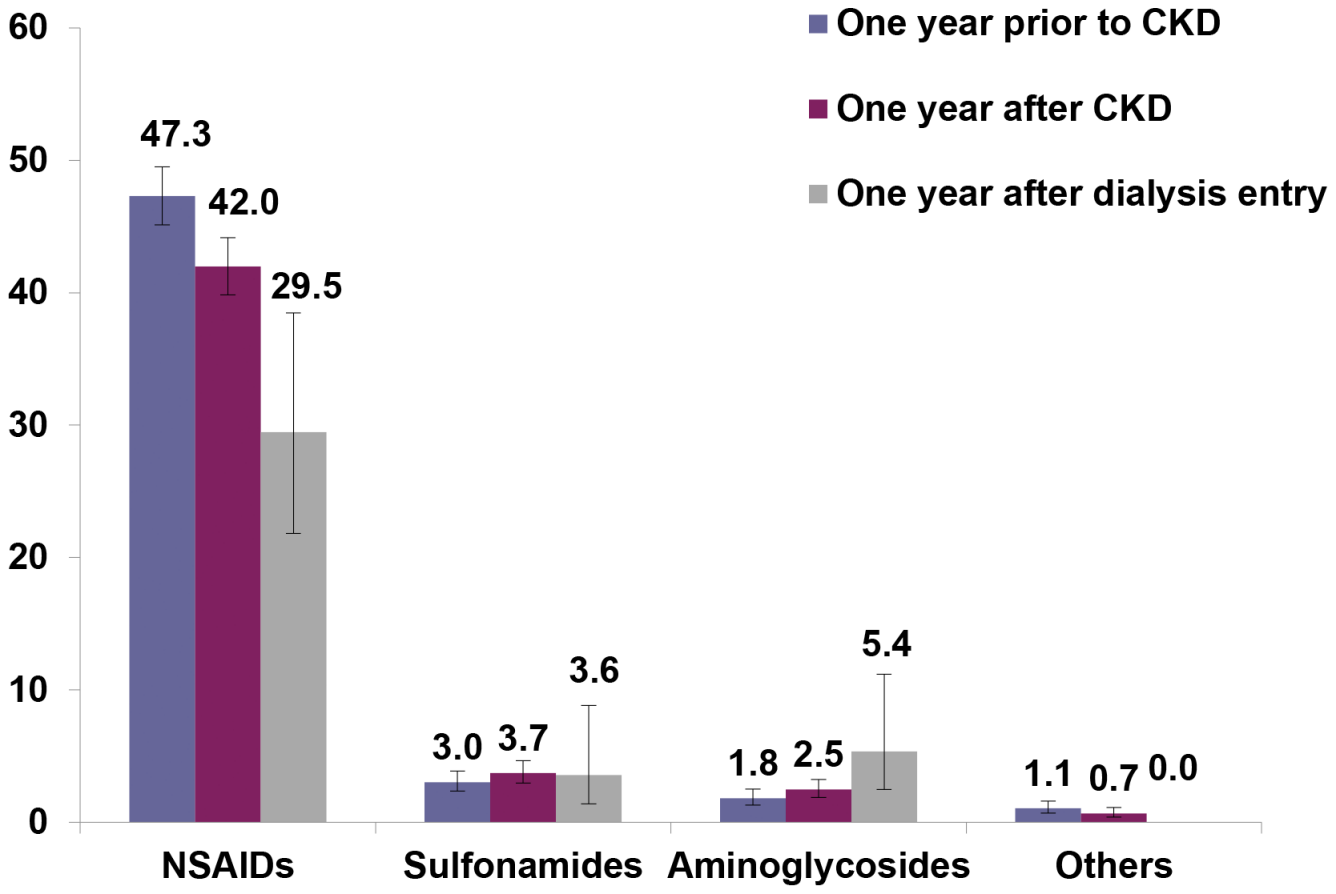
Proportion of CKD patients (N = 1,989) who received ≥ 1 prescription of CND, stratified by classes. Proportion of CKD patients (N = 1,989) who received at least one prescription of contraindicated nephrotoxic drugs, stratified by drug classes, within one year prior or after first CKD diagnosis, and within one year after the entry in dialysis (N = 112) within one year prior or after first CKD diagnosis, and within one year after the entry in dialysis (N = 112). Others: zoledronate, lithium, antineoplastic agents (methotrexate, interferon alfa-2B), gold preparations (auranofin), hydrochlorothiazide. Low dosage acetylsalicylic acid was not included among NSAIDs, but it was analysed in Figure 5, separately. CND: contraindicated nephrotoxic drug.

**Figure 5:**
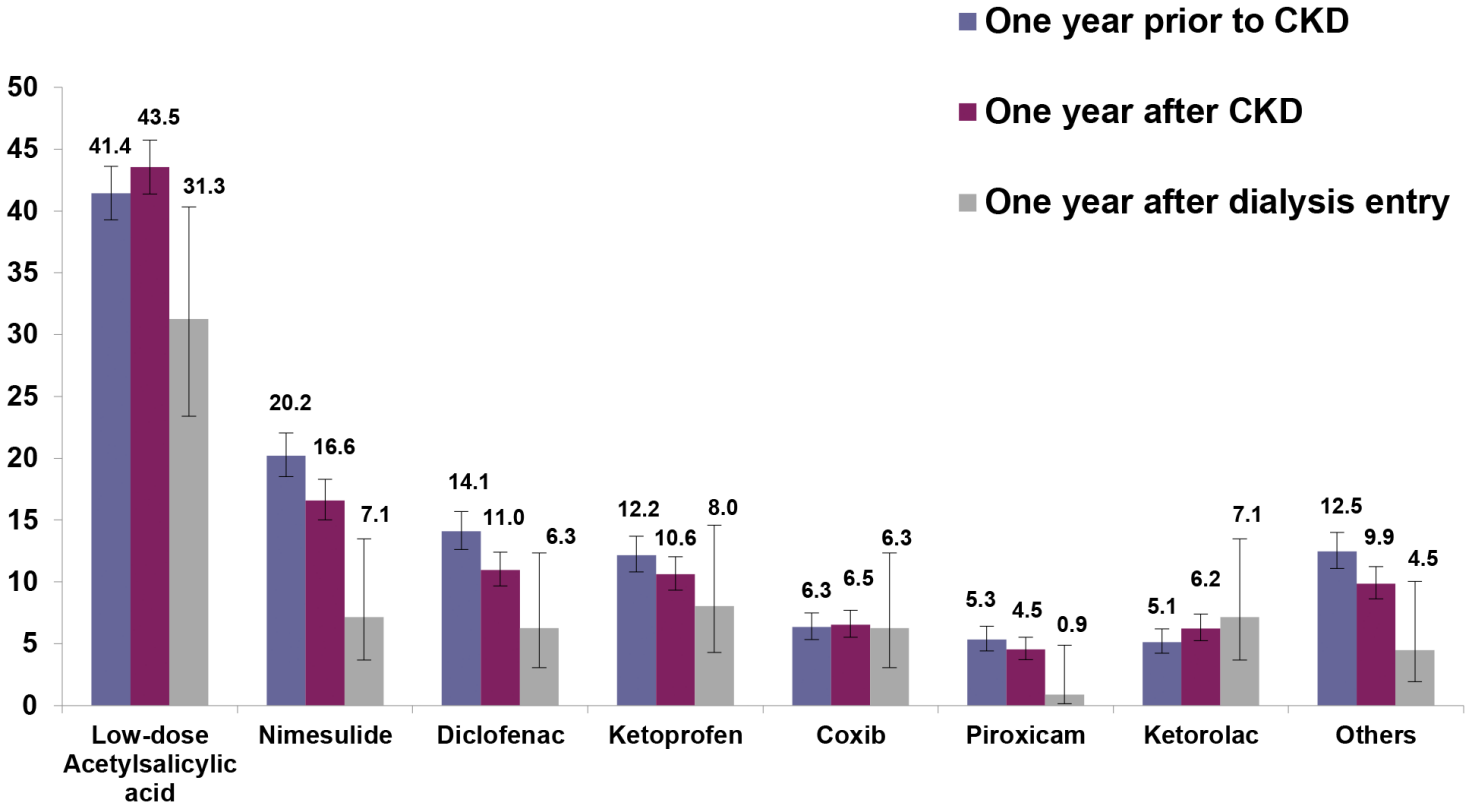
Proportion of CKD patients (N = 1,989) who received ≥ 1 prescription of NSAIDs. Proportion of CKD patients (N = 1,989) who received at least one prescription of individual NSAIDs within one year prior or after the first CKD diagnosis and within one year after the entry in dialysis (N = 112). Others (<5%): tenoxicam, meloxicam, aceclofenac, ibuprofen, dexibuprofen, tiaprofenic acid, naproxen, lornoxicam, acetylsalicylic acid, acetylsalicylic acid and ascorbic acid, niflumic acid, diclofenac sodium plus misoprostol. Coxib: celecoxib, etoricoxib.

Looking at individual drug classes, NSAIDs were the most commonly prescribed contraindicated nephrotoxic drugs to CKD patients during the study period, with a small decrease in the use from one year prior to one year after first CKD diagnosis, and much larger decrease after dialysis entry (47.3% vs. 42.0% vs. 29.5%) (Figure 4). The opposite trend was observed for the use of aminoglycosides (1.8% vs. 2.5% vs. 5.4%), which were in general much less prescribed as compared to NSAIDs (Figure 4). Among NSAIDs used in CKD patients, nimesulide, diclofenac and ibuprofen were the most frequently prescribed compounds, which all showed a small reduction in the use within the first year after CKD diagnosis, as compared to the previous year (Figure 5). A very large proportion of CKD patients received low dosage acetylsalicylic acid and this proportion was even higher after CKD diagnosis (43.5% vs. 41.4%). Overall, 1,119 CKD patients (56.3%) had at least one prescription of any NSAID between the index date and the end of follow-up. Of these, more than one third (35.6%) were treated with NSAIDs for periods exceeding 90 days and almost 16.5% for more than 6 months.

CKD stage-specific codes were reported for 518 (26.0%) CKD patients and in a sensitivity analysis, the 52% of CKD patients at stage IV and V received at least one prescription of contraindicated drug.

The most common reason for starting NSAID therapy was osteoarticular disease (mainly osteoarthrosis, arthralgia, rheumatoid arthritis) with 91.4% of NSAID users in long-term therapy (≥ 6 months) and 77.4% in short-term therapy (< 6 months) having this indication. Table 1 shows the characteristics of incident CKD patients who received short-term (< 6 months) or long-term therapy (≥ 6 months) with NSAIDs as compared to non-users, along with results from logistic regressions. NSAIDs were more likely to be used by female (65.4%) than male (34.6%) CKD patients who received the long-term therapy. Moreover, age-sex adjusted logistic regression showed an OR = 0.48 with 95% CI: 0.34–0.67. CKD patients receiving long-term therapy with NSAIDs were more likely to be between 65–80 years old as compared to <45 years old (Adjusted OR: 15.7; 95% CI: 2.2–114.9) and to be affected by osteoarticular diseases in general and by osteoarthritis in particular, as compared to non-users of NSAIDs. On the contrary CKD patients receiving both short and long -term therapy with NSAIDs were less likely to be affected by liver diseases (Adjusted OR: 0.53; 95% CI: 0.36–0.78 and OR: 0.49; 95% CI: 0.23–1.04 respectively).

**Table 1:** Characteristics of incident CKD patients, stratified by cumulative use of NSAIDs during the follow-up, and results from ordinary and cumulative logistic regressions.

	Non-use of NSAIDs N = 870 (%)	< 180 days of NSAID use N = 934 (%)	Adj. OR* (95% CI) <180 days of NSAID use vs. non-use	≥ 180 days of NSAID use N = 185 (%)	Adj. OR*(95% CI) ≥180 days of NSAID use vs. non-use	Cumulative logistic regression*
**Sex**						
Male	456 (52.4)	460 (49.3)	0.88(0.73–1.06)	64 (34.6)	0.48(0.34 –0.67)	0.74 (0.63–0.88)
Female	414 (47.6)	474 (50.7)	1	121 (65.4)	1	1
**Mean age ± SD**	72.6 ± 15.0	72.1 ± 12.3		72.2 ± 10.0		
**Mean duration of follow-up (years)**	1.8 (0 – 5.9)	3.0 (0 – 6.0)		4.0 (0.6 – 6.0)		
**Age categories**						
<45	55 (6.3)	26 (2.8)	1	1 (0.5)	1	1
45–64	148 (17.1)	197 (21.1)	2.81 (1.67 –4.7)	40 (21.6)	14.86 (1.99–110.75)	3.3 (2.03–5.49)
65–80	357 (41.0)	474 (50.7)	2.81(1.73 –4.57)	102 (55.1)	15.71 (2.19 –114.94)	3.39 (2.11–5.45)
>80	310 (35.6)	237 (25.4)	1.62 (0.98–2.66)	42 (22.8)	7.45 (1.00–55.27)	1.92 (1.18–3.13)
**Comorbidities^1^**						
Diabetes mellitus	296 (34)	358 (38.3)	1.20 (0.99–1.46)	71 (38.4)	1.15 (0.82–1.60)	1.17 (0.98–1.39)
Hypertension	736 (84.6)	819 (87.7)	1.39 (1.04–1.85)	159 (85.9)	1.09 (0.66–1.78)	1.26 (0.97–1.64)
Ischemicheartdisease	329 (37.8)	357 (38.2)	1.05 (0.86–1.27)	48 (25.9)	0.59 (0.41–0.85)	0.89 (0.74–1.06)
Cerebrovascular disease	153 (17.6)	133 (14.2)	0.78 (0.61–1.01)	26 (14.1)	0.75 (0.47–1.19)	0.79 (0.62–1.00)
Liver disease	76 (8.7)	45 (4.8)	0.53 (0.36–0.78)	8 (4.3)	0.49 (0.23–1.04)	0.53 (0.37–0.76)
Malignant neoplasm	101 (11.6)	101 (10.8)	0.95 (0.71–1.27)	10 (5.4)	0.46 (0.24–0.91)	0.82 (0.62–1.08)
Rheumatoid arthritis	13 (1.5)	21 (2.2)	1.50 (0.75–3.02)	8 (4.3)	2.78 (1.12–6.90)	1.89 (1.05–3.41)
Osteoarthritis	443 (50.9)	675 (72.3)	2.74 (2.23–3.36)	156 (84.3)	5.75 (3.68–8.97)	3.04 (2.51–3.69)
Enthesopathies	6 (0.7)	5 (0.5)	0.77 (0.23–2.53)	3 (1.6)	2.52 (0.61–10.41)	1.36 (0.49–3.75)
Gout	255 (29.3)	250 (26.8)	0.92 (0.72–1.18)	36 (19.4)	0.58 (0.35–0.96)	0.82 (0.65–1.04)
Dyslipidemia	251 (28.8)	319 (34.1)	1.29 (1.05–1.57)	54 (29.2)	1.01 (0.71–1.44)	1.18 (0.98–1.41)
**Concomitant drugs^1^**						
Corticosteroids	264 (30.3)	305 (32.7)	1.13 (0.92–1.38)	65 (35.1)	1.27 (0.91–1.79)	1.16 (0.96–1.39)
Vitamin A and/or D†	92 (10.6)	111 (11.9)	1.10 (0.82–1.49)	25 (13.5)	1.07 (0.66–1.75)	1.08 (0.82–1.41)
Opioids	134 (15.4)	135 (14.5)	0.92 (0.71–1.20)	27 (14.6)	0.85 (0.54–1.35)	0.91 (0.72–1.16)

SD =  standard deviation; 95% CI  =  confidence interval; adj. OR =  adjusted OR; mean duration of follow-up (years): mean follow-up between date of incident CKD diagnosis (i.e. index date) and end of the follow-up.

*The analysis was adjusted by sex and age.

^1^ Co-morbidities and concomitant drugs have been evaluated any time prior to the index date and they are not mutually exclusive.

† Combination therapy consisting of vitamin A and D (ATC: A11CB) or vitamin D/vitamin D analogues (ATC: A11CC).

## Discussion

To our knowledge, this is the first population-based study exploring the use of nephrotoxic drugs in CKD patients in Italy. Our study shows that, although contraindicated, such nephrotoxic drugs (mostly NSAIDs) were highly prescribed to CKD patients from a general population of Southern Italy. The new diagnosis of CKD did not seem to reduce the prescription of potentially harmful nephrotoxic drugs, since an elevated number of patients continued to receive prescriptions of nephrotoxic drugs after CKD diagnosis. Substantial variability in the use of nephrotoxic drugs among 123 GPs was observed. Nimesulide was found to be the most commonly prescribed NSAIDs.

In general, use of contraindicated drugs exposes CKD patients to a high risk of worsening of renal function. The prescribing pattern of contraindicated nephrotoxic drugs was not substantially modified after CKD diagnosis or after dialysis entry. This may be justified because renal function is mostly lost by the time dialysis is required and is completely supplanted by the dialysis process. However, avoiding the use of nephrotoxic drugs such as NSAIDs, aminoglycosides etc. is not only a predialytic measure aimed at preventing kidney disease progression, but should also be a post-dialysis measured to preserve residual renal function [24].

An Australian longitudinal cohort study with 3,175 persons >18 years from the general population showed high use of NSAIDs in patients with renal disease as 31% of users of these drugs had stage 3 or higher CKD stage [25], similar to our study findings. Several nephrotoxic drugs, including NSAIDs, are known to exert nephrotoxic effects through renal vasoconstriction and clinically significant reduction in glomerular filtration rate (GFR) via renal prostaglandin inhibition, or through other mechanisms as occurs in interstitial nephritis, membranous glomerulonephropathy, type 4 renal tubular acidosis and acute and chronic renal papillary necrosis [26]–[28]. Long-acting NSAIDs or those having a half-life >12 hours should be avoided to prevent persistent and clinically significant GFR reduction induced by NSAIDs via inhibition of renal vasodilatory prostaglandins [29].

The most common reason for starting therapy with NSAIDs, in our study cohort, was the occurrence of osteoarticular disease (mainly osteoarthrosis, arthralgia, rheumatoid arthritis) with 91.4% of NSAID users in long-term therapy (≥ 6 months) having this indication. Pain, a common complaint in osteoarticular diseases [30], has been reported to be a common problem in end-stage renal disease (ESRD) patients [31]. Moreover, the overuse of over-the-counter NSAIDs is particularly common in rheumatic conditions such as osteoarthritis [32], thus further highlighting the importance of our results in CKD patients with such diseases who are already use prescription-only NSAIDs. Pain treatment in CKD still remains a primary issue for clinicians: while, in general, acetaminophen has been considered to be the safest non-narcotic analgesic in CKD patients it may be nephrotoxic with chronic use at high doses [33]. For treatment of moderate pain, the use of low-potency opioids is suggested unless CKD is very advanced [34]. Tramadol may be also used because it is not known to be nephrotoxic. Nonetheless, it must be noted that its systemic elimination is reduced with advanced CKD, thus requiring dose adjustment in patients with renal disease. Opioids without potential for accumulation such as fentanyl, buprenorphine and hydromorphone can be considered as valid option in case of severe pain [35], [36].

Our results also show that low-dose acetylsalicylic acid for cardiovascular prevention is widely prescribed in elderly patients with CKD [37]. According to the Summary of Product Characteristics, low-dose acetylsalicylic acid is contraindicated in patients with severe CKD (GFR <30 ml/min). However, evidence suggests that early CKD increases risk of cardiovascular events and death [38]–[40]. As a result, the absolute benefits of aspirin might be greater for CKD patients than the risk of worsening kidney function. On the other hand, CKD patients have abnormal platelet function, potentially leaving them at increased risk of hemorrhage when treated with anticoagulants or antiplatelet agents [41]. A meta-analysis of cardiovascular risk reduction with aspirin therapy suggests that aspirin reduces cardiovascular risk, both in primary and secondary prevention, but the protective effect on patients with renal dysfunction was unknown [42]. A randomised-controlled trial on 18,597 hypertensive patients investigating associations between the benefits of aspirin and glomerular filtration found that aspirin therapy prevented significantly more cardiovascular events and deaths, and all-cause deaths in CKD patients compared to those with normal kidney function [43]. Major cardiovascular events were reduced by 9%, 15% and 66% for patients with mild, moderate, and severe CKD respectively, even though the risk of major or minor bleeding in CKD subgroups was nearly 10 times higher than the general population. The overall benefits of aspirin seem to outweigh the risk of bleeding [44]. Finally, our findings show also a substantial increase in CKD patients treated with aminoglycosides after dialysis entry (5.4%), probably due to an increase in infections following use of central venous catheters or arteriovenous fistula for renal hemodialysis.

This study has several strengths as well as limitations. We included CKD patients on dialysis in our study, unlike other similar studies [45], which allowed us to investigate the prescription of nephrotoxic drugs even in these patients. In addition, the methodology used here has the advantages of an administrative database study: it is free from biases such as recall and selection bias. However, diagnoses recorded by GPs may not be accurate despite we used very specific codes for renal disease and related procedures. We cannot exclude a possible misclassification of acute kidney injury (AKI) as chronic kidney disease (CKD). However, for case ascertainment we considered only specific CKD codes and in presence of less specific codes, we considered these codes only if registered multiple times to ensure the disease was chronic. We cannot identify the exact date of onset of CKD as it was defined as the date of first registration of CKD-related primary/secondary hospital discharge diagnosis, procedure or indication of use for drug prescription. Nonetheless, the frequency of use of nephrotoxic drugs after CKD diagnosis is very high and only slightly lower as compared to the year prior to the diagnosis, thus confirming poor awareness of prescribers about possible detrimental effects of these drugs in CKD patients. Some drugs are specifically contraindicated in patients with severe renal disease (creatinine clearance < 30 ml/min) only. We could not identify the stage for most CKD patients due to lack of laboratory data (e.g. serum creatinine test, GFR value) and registration of CKD codes of unspecified stage. For this reason the rate of contraindicated nephrotoxic drug use may have been overestimated. However, CKD stage-specific codes were reported for 518 (26.0%) CKD patients and in a sensitivity analysis restricted to CKD stage IV and V patients, similar figures concerning contraindicated drug use were observed. We used outpatient prescription data and we had no information about actual filling of prescriptions and medication use. However, this study was primarily aimed at exploring the prescribing pattern of nephrotoxic drugs and it is unlikely that NSAIDs, generally prescribed as painkiller, were not ultimately taken by the patient. Furthermore, the traceability of some nephrotoxic drug prescriptions was incomplete as being administered in the hospital (e.g. oncology drugs, and partly, aminoglycosides) or used as OTC drugs (e.g. NSAIDs), as a consequence, the nephrotoxic drugs use could be underestimate. In addition, this study was carried out using outpatient data collected from a large general practice of Southern Italy. We therefore cannot exclude that these findings are not fully generalizable to the whole Italian general population. However, the applied methodology and the Arianna database have been shown to provide accurate and reliable information for pharmacoepidemiological research, as documented elsewhere [15]–[21].

## Conclusions

This study shows that contraindicated nephrotoxic drugs (mostly NSAIDS) are frequently prescribed to CKD patients and the new diagnosis of CKD did not modify the prescription pattern. NSAIDs were most commonly used as analgesics in diseases such as osteoarthritis and rheumatic arthritis. Therapeutic alternatives such as acetaminophen and weak opioids can be used at the lowest dosages and shortest durations in patients whose CKD is not advanced. More awareness is needed about the safety issues of using nephrotoxic drugs in CKD. Intervention strategies to improve prescribing pattern in CKD patients should be mostly targeted to GPs who prescribe more frequently contraindicated nephrotoxic drugs.

## Supporting Information

Table S1 **List of contraindicated nephrotoxic drugs in patients with renal disease on the basis of the summary of product characteristics (SPC).**
(DOCX)

Table S2 **List of drugs to be used with caution in patients with renal disease on the basis of the summary of product characteristics (SPC).**
(DOCX)

Table S3 **List of nephrotoxic drugs classified by CKD stage-specific contraindication as reported in Summary of Product Characteristics (SPC).** CKD: chronic kidney disease; BUN: Blood Urea Nitrogen; GFR: glomerular filtration rate.
(DOCX)

Table S4 **Diagnosis or procedure codes to identify CKD and dialyzed patients.** Patients identified through ICD9-CM code “583*” or “586*” were considered as CKD patients only if these codes were repeated more than twice during the study period to prevent the misclassification of patients with acute renal disease as CKD.
(DOCX)
