# Supplementary material for: The Burden of Nephrotoxic Drug Prescriptions in Patients with Chronic Kidney Disease: A Retrospective Population-Based Study in Southern Italy
Source: PLoS One. 2014 Feb 18;9(2):e89072. doi: 10.1371/journal.pone.0089072 (PMC3928406; doi:10.1371/journal.pone.0089072)
Supplement: Table S1 — List of contraindicated nephrotoxic drugs in patients with renal disease on the basis of the summary of product characteristics (SPC). (DOCX) [file pone.0089072.s001.docx]

**Table S1.** List of contraindicated nephrotoxic drugs in patients with renal disease on the basis of the summary of product characteristics (SPC).

| **Contraindicated drugs** | |
| --- | --- |
| **NSAIDs** | **Gold Preparations** |
| Diclofenac | Auranofin |
| Ketorolac | Sodiumaurotiosulphate |
| Aceclofenac | **Thiazides** |
| Piroxicam | Hydrochlorothiazide |
| Tenoxicam | **Sulfonamides** |
| Lornoxicam | Sulfamethoxazole and trimethoprim (combination therapy) |
| Meloxicam | Sulfametrole and trimethoprim (combination therapy) |
| Ibuprofen | Sulfamazone |
| Naproxen | **Aminoglycosides** |
| Ketoprofen | Streptomycin |
| Celecoxib | Tobramycin |
| Etoricoxib | Gentamicin |
| Acetylsalicylic acid | Amikacin |
| Etodolac | Netilmicin |
| Acemetacin | **Antineoplastic agents** |
| Naproxen and Esomeprazol (combination therapy) | Methotrexate |
| Dexketoprofen | Interferon alfa-2b |
| Rofecoxib | Cisplatin |
| Diclofenac sodium and misoprostol (combination therapy) | **Others** |
| Tiaprofenic acid | Zoledronate |
| Dexibuprofen | Lithium |
| Niflumic acid | Colistin |
| Nimesulide |  |
| Acetylsalicylic acid and ascorbic acid (combination therapy) |  |
| Low-dose aspirin |  |
