# Supplementary material for: The Burden of Nephrotoxic Drug Prescriptions in Patients with Chronic Kidney Disease: A Retrospective Population-Based Study in Southern Italy
Source: PLoS One. 2014 Feb 18;9(2):e89072. doi: 10.1371/journal.pone.0089072 (PMC3928406; doi:10.1371/journal.pone.0089072)
Supplement: Table S2 — List of drugs to be used with caution in patients with renal disease on the basis of the summary of product characteristics (SPC). (DOCX) [file pone.0089072.s002.docx]

**Table S2.** List of drugs to be used with caution in patients with renal disease on the basis of the summary of product characteristics (SPC)

| **Drugs to be used with caution** | | |
| --- | --- | --- |
| **Angiotensin-converting enzyme inhibitors (ACEI)** | **Cephalosporins** | **Quinolones** |
| Captopril | Cefalexin | Ofloxacin |
| Enalapril | Cefalotin | Ciprofloxacin |
| Lisinopril | Cefazolin | Pefloxacin |
| Ramipril | Cefadroxil | Enoxacin |
| Quinapril | Cefatrizine | Norfloxacin |
| Benazepril | Cefradine | Lomefloxacin |
| Fosinopril | Ceftezole | Rufloxacin |
| Trandolapril | Cefuroxime | Levofloxacin |
| Spirapril | Cefaclor | Moxifloxacin |
| Moexipril | Cefonicid | Prulifloxacin |
| Zofenopril | Cefmetazole | Nalidixic acid |
| Delapril | Cefprozil | Pipemidic acid |
| Cilazapril | Cefoxitin | Cinoxacin |
| Perindopril | Cefamandole | **Antivirals for systemic use** |
| Clopidogrel | Cefotetan | Aciclovir |
| **Penicillins** | Cefotaxime | Tenofovir |
| Ampicillin | Ceftazidime | Adefovir |
| Amoxicillin | Ceftriaxone | Foscarnet |
| Bacampicillin | Ceftizoxime | Ganciclovir |
| Piperacillin | Cefixime | Indinavir |
| Benzathinebenzylpenicillin | Cefodizime | **Others** |
| Flucloxacillin | Cefoperazone | Amphotericin B |
| Ampicillin and enzyme inhibitor | Cefpodoxime | Quinine |
| Piperacillin and enzyme inhibitor | Ceftibuten | Vancomycin |
| Benzylpenicillin | Cefditoren | Pentamidine |
| [**Angiotensin II antagonists**](http://www.whocc.no/atc_ddd_index/?code=C09CA&showdescription=no) | Cefetamet | Pamidronate |
| Valsartan | Cefepime | Allopurinol |
| Irbesartan | **Calcineurin inhibitors** | Ranitidine |
| Candesartan | Ciclosporine |  |
| Telmisartan | Tacrolimus |  |
| Olmesartan |  |  |
| Losartan |  |  |
