# Supplementary material for: The Burden of Nephrotoxic Drug Prescriptions in Patients with Chronic Kidney Disease: A Retrospective Population-Based Study in Southern Italy
Source: PLoS One. 2014 Feb 18;9(2):e89072. doi: 10.1371/journal.pone.0089072 (PMC3928406; doi:10.1371/journal.pone.0089072)
Supplement: Table S3 — List of nephrotoxic drugs classified by CKD stage-specific contraindication as reported in Summary of Product Characteristics (SPC). CKD: chronic kidney disease; BUN: Blood Urea Nitrogen; GFR: glomerular filtration rate. (DOCX) [file pone.0089072.s003.docx]

**Table S3.** List of nephrotoxic drugs classified by CKD stage-specific contraindication as reported in Summary of Product Characteristics (SPC)

|  | | |  |  |
| --- | --- | --- | --- | --- |
| **Moderate kidney disease** | **Severe kidney disease** | **Not specified** | | **Notes** |
| Ketorolac | Ketorolac |  | | “Serum creatinine>1.8mg/dl” |
|  | Diclofenac |  | |  |
|  | Diclofenac sodium and misoprostol (in combination) |  | |  |
|  | Piroxicam |  | | “Severe renal disorders” |
|  | Meloxicam |  | | “Only severe CKD, patients not undergoing dialysis” |
|  | Ibuprofen |  | |  |
|  | Etoricoxib |  | | “Creatinine clearance <30 ml/min” |
|  | Ketoprofen |  | |  |
|  |  | Naproxen (I.V. formulation) | | “Renal hypoperfusion, renal disease” |
|  | Acetylsalicylic acid |  | |  |
|  | Acetylsalicylic acid and ascorbic acid (in combination) |  | |  |
|  | Celecoxib |  | | “Creatinine clearance <30 ml/min” |
|  | Low-dose acetylsalicylic acid |  | |  |
|  | Aceclofenac |  | |  |
|  | Tenoxicam |  | | “Severe renal disorders” |
|  | Lornoxicam |  | |  |
|  | Etodolac |  | |  |
|  | Acemetacin |  | | “Severe renal disorders” |
|  | Tiaprofenic acid |  | |  |
|  | Dexibuprofen |  | | “Severe renal disorders (GFR<30 ml/min)” |
|  | Dexketoprofen |  | |  |
|  | Naproxen and esomeprazol (in combination) |  | |  |
|  | Rofecoxib |  | | “Creatinine clearance <30 ml/min” |
|  | Niflumic acid |  | |  |
|  | Nimesulide |  | |  |
|  | Sulfamethoxazole and trimetoprim(in combination) |  | | “Severe renal failure with BUN” |
|  | Sulfametrole and  Trimetoprim (in combination) |  | | “Severe renal failure with BUN” |
|  | Sulfamazone |  | | “Severe renal failure with BUN” |
|  | Hydrochlorothiazide |  | | “Creatinine clearance <30 ml/min” |
|  | Gentamicin (I.V. formulation) |  | |  |
|  |  | Streptomycin | | “Renal failure” |
|  | Zoledronic acid |  | | “Acute renal impairment and in patients with a creatinine clearance of less than 35 ml/min or renal failure” |
|  |  | Lithium | | “Renal failure” |
|  | Methotrexate |  | | “Renal failure (creatinine clearance <20 ml/min)” |
|  | Interferon alfa-2b |  | |  |
| Cisplatin |  |  | | “Renal disease (creatinine clearance <60 ml/min)” |
|  |  | Auranofin | | “Progressive renal failure” |
|  |  | Sodiumaurotiosulphate | | “Renal failure” |
|  |  | Colistin | | “Renal failure” |

**Legend**: CKD: chronic kidney disease; BUN: Blood Urea Nitrogen; GFR: glomerular filtration rate
