# Supplementary material for: The Burden of Nephrotoxic Drug Prescriptions in Patients with Chronic Kidney Disease: A Retrospective Population-Based Study in Southern Italy
Source: PLoS One. 2014 Feb 18;9(2):e89072. doi: 10.1371/journal.pone.0089072 (PMC3928406; doi:10.1371/journal.pone.0089072)
Supplement: Table S4 — Diagnosis or procedure codes to identify CKD and dialyzed patients. Patients identified through ICD9-CM code “583*” or “586*” were considered as CKD patients only if these codes were repeated more than twice during the study period to prevent the misclassification of patients with acute renal disease as CKD. (DOCX) [file pone.0089072.s004.docx]

**Table S4.** Diagnosis or procedure codes to identify CKD and dialyzed patients.

|  | **CKD codes** | **Dialysis codes** |
| --- | --- | --- |
| **Diagnosis** | 250.4 (diabetes with renal manifestations) | 585.6 (chronic kidney disease, stage V requiring chronic dialysis) |
|  | 285.21 (anemia in chronic kidney disease) | 792.5 (cloudy (hemodialysis) (peritoneal) dialysis effluent) |
|  | 583* (nephritis and nephropathy, not specified as acute or chronic)^ | 996.1 (mechanical complications involving aortic (bifurcation) graft (replacement) arteriovenous: dialysis catheter) |
|  | 585* (chronic kidney disease) | 996.56 (mechanical complication of other specified prosthetic device, implant, and graft - due to peritoneal dialysis catheter) |
|  | 586* (renal failure, unspecified)^ | 996.68 (infection and inflammatory reaction due to internal prosthetic device, implant, and graft - Due to peritoneal dialysis catheter) |
|  | 996.73 (other complications of internal (biological) (synthetic) prosthetic device, implant, and graft - due to renal dialysis device, implant, and graft) | 996.73 (other complications of internal (biological) (synthetic) prosthetic device, implant, and graft - Due to renal dialysis device, implant, and graft) |
|  | V45.1* (renal dialysis status) | V45.1* (renal dialysis status) |
|  | V56* (encounter for dialysis and dialysis catheter care) | V56* (encounter for dialysis and dialysis catheter care) |
|  | 403* (hypertensive chronic kidney disease) | E870.2 (accidental cut, puncture, perforation, or hemorrhage during medical care: Kidney dialysis or other perfusion) |
|  | 404* (hypertensive heart and chronic kidney disease) | E871.2 (foreign object left in body during procedure: Kidney dialysis or other perfusion) |
|  |  | E872.2 (failure of sterile precautions during procedure: Kidney dialysis and other perfusion) |
|  |  | E879.1 (kidney dialysis) |
| **Procedures** | 39.27(arteriovenostomy for renal dialysis) | 39.27 (arteriovenostomy for renal dialysis) |
|  | 55.23 (closed [percutaneous] [needle] biopsy of kidney) | 38.95 (venous catheterization for renal dialysis) |
|  | 55.24 (open biopsy of kidney) | 39.42 (revision of arteriovenous shunt for renal dialysis) |
|  |  | 39.43 (removal of arteriovenous shunt for renal dialysis) |
|  |  | 39.95 (hemodialysis) |
|  |  | 54.98 (peritoneal dialysis) |

^Patients identified through ICD9-CM code“583*” or “586*” were considered as CKD patients only if these codes were repeated more than twice during the study period to prevent the misclassification of patients with acute renal disease as CKD.
